# 

**Published:** 1824-07

**Authors:** 


### THE LONDON MEDICAL AND PHYSICAL JOURNAL.

CONTAINING

ORIGINAL CORRESPONDENCE OF EMINENT PRACTITIONERS,

AND

CRITICAL ANALYSIS OF NEW WORKS

RELATING TO MEDICINE, SURGERY, MIDWIFERY, CHEMISTRY, PHARMACY, BOTANY, AND NATURAL HISTORY.

---

EDITED BY

RODERICK MACLEOD, M.D.

LICENTIATE OF THE COLLEGE OF PHYSICIANS OF LONDON; MEMBER OF THE MEDICO-CHIRURGICAL SOCIETY OF LONDON, AND OF THE ROYAL MEDICAL SOCIETY OF EDINBURGH; PHYSICIAN TO THE WESTMINSTER GENERAL DISPENSARY; TO THE INFIRMARY FOR CHILDREN, AND TO THE SCOTTISH HOSPITAL; LECTURER ON THE PRACTICE OF PHYSIC AND MATERIA MEDICA, AT THE MEDICAL THEATRE, WINDMILL-STREET:

AND

JOHN BACOT, ESQ.

MEMBER OF THE ROYAL COLLEGE OF SURGEONS; SURGEON TO THE ST. GEORGE'S AND ST. JAMES'S DISPENSARY; AND LATELY SURGEON TO HIS MAJESTY'S GRENADIER REGIMENT OF FOOT GUARDS.

---

VOL. LII.

FROM JULY TO DECEMBER, 1824.

Et quoniam variant morbi, variabimus artes;

Mille mali species, mille salutis erunt.

---

45585

*LONDON:*

PRINTED FOR THE PROPRIETORS,

*By J. and C. Adlard, Bartholomew Close*;

PUBLISHED BY J. SOUTER, 73, ST. PAUL'S CHURCH-YARD; AND MAY BE HAD OF ALL BOOKSELLERS.

W1
LD 31

### INDEX

OF

THE FIFTY-SECOND VOLUME

OF THE

### London Medical and Physical Journal.

PAGE

ABDOMINAL abscess, case of . 278
Acaris, a new species of . 527
Account of a man who swallowed a knife . 397
Acid, sulphureous, liquification of 178
——, hydrocyanic, in Tænia . 530
After-pains, on . 291
Affection of the brain, tuberculous 529
Agents, physical, on the influence of, on animal life . 20
Air in the veins, death from . 525
Alteration of solutions by air . 179
Amputation of the hip, observations on . 66
—— of the lower jaw . 68
——, remarks on . 74
—— of the shoulder-joint . 76
Anatomy of the ear, on . 3
——, comparative system of . 8
——, pathological, of the peritoneum . 37
Analysis of the Male Fern-root . 264
—— Upas . 264, 442
Anæmia, case of . 51
Anasarca during pregnancy . 439
Apoplexy, on the influence of the stomach in producing . 350
Appearances of the stomach, on certain . 457
Arteries, on the surgical anatomy of 9
Artificial chalybeate water . 178
Arachnoid membrane, ossifications of . 259
Ascites during pregnancy . 302

Bad effects of Ergot . 530
Belladonna, preventive of Scarlet Fever . 57, 262
Bladder, rupture of the . 259
Blood, on the condition of, in disease . 43
——, on the . 45
—— in Jaundice, on the . 258
Bowels, on obstruction of . 61
Brain, remarks on . 9
—— on the circulation in the . 18
—— of fishes, on the . 17

PAGE

Brain, bifid, remarks on . 24
—— diseased, cases of . 30
—— tuberculous affection of . 529
Bronchiæ, alterations of . 175
——, diseases of, on the diagnosis 432

Case of Anœmia . 51
—— formation of pus in the stomach . 123
—— wound in dissection . 125
—— Abdominal Abscess . 278
—— Rupture of the Bladder . 259
—— Hydrocephalus cured by puncture . 261
—— Gangrene of the Heart . 35
—— diseased Brain . 30
—— extirpation of the Parotid Gland . 63
—— amputation of the Lower Jaw 68
—— Wound through the Diaphragm . 260
—— Chronic Hydrocephalus . 462
—— interruption of the Spinal Chord . 31
—— tuberculous affection of the Brain . 529
—— Diplopia . 178
—— Ascites during Pregnancy 302
—— Puerperal Fever . 214
—— Lithotomy performed on a Horse . 304
—— Anasarca during Pregnancy 439
—— extirpation of a large Tumor 72
—— Rupture of the Heart . 528
—— spontaneous Evolution of the Fœtus . 211
—— Laryngitis . 218
—— Rupture of the Vena Cava 527
Cases of Cholera . 382
—— Chorea . 129, 216
—— Malformation . 34, 367
—— Neuralgia . 373
Caoutchouc, on the preparation of 444
Capsules, on the supra-renal . 7
Castor Oil, on a substitute for . 531
Castration, on the effects of 259
Cæsarean operation, substitute for 436

PAGE
Cellular Membrane, on diffuse inflammation of . . . 40
Certain appearances of the Stomach 451
Chalybeate water, artificial . 178
Chick in ovo, on the sex of . 486
Cholera of India, on the . . 176
———, remarks on . . 295
Chorea, Nitrate of Silver in . 262
Comparative anatomy, system of . 8
Contagious Glanders, on . . 444
Croton Oil, on . . . 435
Cure of Palsy, spontaneous . 352
Cynanche Trachealis, on . . 298

Death from the introduction of air into the veins . . . 525
Detection of Hydrocyanic Acid in the body . . . 263
Diagnosis of disease of the Bronchiæ 432
Diaphragm, wound through the . 260
Diffuse inflammation of the cellular membrane . . . 40
Digitaline, observations on . 441
Diplopia, case of . . . 178
Diseased Brain, cases of . . 30
Diseases, putrid, on . . 52
Dissection, case of wound from . 125
Dropsy, remarks on . . 447

Ear, on the anatomy of . . 3
Effect of lightning . . . 393
Effects of castration, on the . 259
Elongation of the lower extremities 260
Enteritis, remarks on . . 116
Epilepsy, observations on . . 309
———, Mugwort a remedy for . 531
———, Tartar-emetic ointment in 306
Ergot, on the bad effects of . 530
Exhalation of water from the Lungs 176
Experiments, remarks on M. Magendie's . . . . 95
——— on the Itch . . 437
——— Nerves . 82
——— Vomiting . 524
Extirpation of the Parotid Gland 63
Eye, on a new muscle of the . 2

Fern-root, male, analysis of . 264
Fishes, on the brain of . . 17
Fœtus, on the spontaneous evolution of . . . . 211
Fracture-bed, remarks on Mr. Earle's 375
Fractures, application of the stethoscope to . . . 77
Fumigation, remarks on . 387, 490
Functions of certain nerves, on . 15

Gall-bladder, on the structure of . 258
Gangrene of the Heart, case of . 35
Glanders, contagious, on . . 444
Glandular structure in the uterus of certain animals . . 5

PAGE
Heart, diseases of the . . 33
———, gangrene of the . . 35
———, on the rupture of . 175, 528
Hemorrhage, uterine, on . . 440
———, on Nitrate of Potash in 531
Hernia, observations on . 378, 369
Homœopathia, on . . . 56
Horse, Lithotomy performed on . 304
Hydrocephalus cured by puncture 261
———, chronic, case of . 462
———, remarks on . 191
Hydrocyanic acid, remarks on, 63, 262
———, on the detection of . . . . 263
——— in Tænia . 530
Hydrophobia, pustules under the tongue in . . . 526
———, observations on . 433

Infanticide, remarks on . . 264
Inflammation, diffuse, of the Cellular Membrane . - . 40
——— of the Spinal Marrow 17
India, on the Cholera of . 176, 295
Itch, experiments on . . 437
Jaundice, on the blood in . . 258

Knife, man who swallowed, account of . . . . 397

Laryngitis, case of . . 218
Lightning, effects of . . 393
Liquifaction of Sulphureous Acid 178
Lithotomy, new method of performing . . . . 263
——— performed on a horse . 304
Lower extremities, on the elongation of . . . . 260
——— Jaw, amputation of . 68
Lunacy, on pumping in cases of . 443
Lungs, exhalation of water from . 176
Lying-in Hospital at Florence, statements relating to . . 438

Malformation, cases of . 34, 367
Malta, on the Topography of . 469
Mammæ, menstruation from . 434
Man who swallowed a knife, account of . . . . 397
Method, new, of performing Lithotomy . . . . 263
Mugwort, a remedy in Epilepsy . 531
Muscle of the Eye, on a new . 2

Nævi, remarks on . . 303
Nervous system, on the . . 11
Nerves, on the functions of . 14, 15
———, optic, on the semi-decussation of . . . . 390
———, experiments on the . 82
Neuralgia, cases of . . 373
———, remarks on . . 281

PAGE
Neuralgia, Oil of Turpentine a remedy for . . . 62
New species of Acaris . . 527
Nitrate of Potass in Hemorrhage 531
———— Silver in Chorea . 262

Observations on amputation at the Hip . . . . 66
———— Cynanche Trachealis 298
———— Digitaline . . 441
———— Epilepsy . . 309
———— Fumigation . 490
———— Hydrophobia . 433
———— Phlegmasia Dolens 434
———— Tartar-emetic ointment ib.
———— the Topography of Malta 469
Obstruction of the Bowels, on . 61
Ocular diseases, report of. 183, 271
Oil of Croton, on . . . 435
——Turpentine, remedy in Neuralgia 62
Ointment, Tartar-emetic, in Epilepsy 306
Operation, substitute for Cæsarean 436
Optic nerves, on the semi-decussation of . . . . 390
————, on the wasting of . 524
Ornythohyncus, on the generative system of . . . 6
Ossifications of the Arachnoid Membrane . . . . 259

Palsy, spontaneous cure of . 352
Parturition, sudden, on . . 353
Parotid Gland, case of extirpation of 63
Pathological anatomy of the Peritoneum . . . . 37
Percussion and compression in Rheumatism . 104, 200, 284
Phlegmasia Dolens, observations on 434
Physical agents, on the influence of 20
Pregnancy, Ascites during . 302
Preparation of Caoutchouc . 444
Puerperal Fever, case of . . 214
Pumping in cases of Lunacy . 443
Pulvis Antimonialis, remarks on 64, 177
Pustules under the tongue in Hydrophobia . . . 526
Putrid diseases, on . . 52
Pyrola Umbellata, on the virtues of 62

Remarks on Amputation . . 74
———— at the Shoulder-joint . . . 76
———— Cholera . . 295
———— Dropsy . . 447
———— Enteritis . . 116
———— on Fumigation . 387
———— Hydrocephalus . 191
———— Hydrocyanic Acid 63, 262
———— Infanticide . . 264
———— Mr. Earle's Fracture-bed 375
———— M. Magendie's experiments 95
———— Neuralgia . . 281

PAGE
Remarks on Pulvis Antimonialis 64, 177
———— Snuff-taking . . 481
———— Syphilis . . 78
Report of ocular diseases - 183, 271
Rheumatism, on compression and percussion in . 104, 200, 284
Rupture of the Bladder, case of 259
———— Heart . 175, 528
———— Vena Cava . 527

Scarlet Fever, on Belladonna as preventive of . . 57, 262
Sex of the Chick in ovo . . 486
Shoulder-joint, amputation at . 76
Small-pox after Vaccination . 208
Snuff-taking, remarks on . . 481
Solutions, alterations of, by air . 179
Spleen, on the contraction of . 23
Spinal Chord, on the interruption of 31
—— Marrow, inflammation of the 17
Spine, curvatures of the . . 359
Spontaneous cure of Palsy . 352
———— evolution of the Fœtus. 211
State of the Blood in disease . 43
Statements relating to the Lying-in Hospital at Florence . 438
Stethoscope, application of, to Fractures . . . 77
Stomach, on diseases of the . 40
————, formation of pus in . 123
————, on the influence of, in producing Apoplexy . . 350
Structure of the Gall-bladder . 258
Substitute for Castor-oil . . 531
Sudden Parturition, on . . 353
Sulphureous Acid, liquifaction of 178
Supra-renal Capsules, on . . 7
Surgical anatomy of Arteries, on . 9
Syphilis, remarks on . . 78
System of Comparative Anatomy . 8

Tartar-emetic ointment in Epilepsy 306
————, on . 434
Tænia, Hydrocyanic Acid in . 530
Topography of Malta, observations on . . . . . 469
Tuberculous affection of the Brain 529
Tumor, large, extirpation of . 72
Turpentine, the Oil of, a remedy in Neuralgia . . . 62

Vaccination, on Small-pox after . 208
Vena Cava, rupture of . . 527
Vomiting, experiments on . 524
Upas, on the analysis of . 264, 442
Uterine Hemorrhage, on . . 440
Uterus, on a glandular structure in certain animals . . 5

Wasting of the Optic Nerves, on . 524
Water, artificial Chalybeate . 178
———, exhalation of, from the Lungs 176

### INDEX.

### REVIEWS.

### DOMESTIC.

PAGE

The Principles of Forensic Medicine, systematically arranged and applied to British Practice. By J. GORDON SMITH, M.D. &c. . . 135, 223

Pathological Remarks on the Rotated and Contorted Spine, commonly called Lateral Curvature, deduced from Practice, &c. By ANDREW DODS, M.D. late of Edinburgh, and Surgeon in the Royal Navy . . 157

An Inquiry into the Causes of the Curvatures of the Spine; with Suggestions as to the means of preventing, or, when formed, of removing, the Lateral Curvature. By T. JARROLD, M.D. . . . . . . 159

An Elementary System of Physiology. By JOHN BOSTOCK, M.D. F.R.S. Vol. I. . . . . . . . . . . . 160

The Animal Kingdom, arranged in conformity with its Organization, by Baron CUVIER. With additional Descriptions of the Species hitherto named, of many not before noticed, and other original Matter, by EDWARD GRIFFITHS, F.L.S. . . . . . . . . . 166

Observations illustrative of the Nature and Treatment of the prevailing Disorders of the Stomach and Liver. By THOMAS JOHN GRAHAM, M.D. Member of the Royal College of Surgeons, London . . . 241

A Practical Treatise on Diseases of the Skin; comprehending an Account of such Facts as have been recorded on these Subjects, with original Observations. The whole arranged with a view to illustrate the Constitutional Causes of these Diseases, as well as their local Character. By SAMUEL PLUMBE, Member of the Royal College of Surgeons . . . 315

A Practical Treatise on Hæmorrhoids or Piles, Strictures, and other important Diseases of the Rectum and Anus; being, with some Additions, a Treatise to which the Jacksonian Prize was adjudged by the Royal College of Surgeons. By GEORGE CALVERT, Member of the Royal College of Surgeons, London, &c. . . . . . . . . 332

Concise Narrative of an Ophthalmia which prevailed in a Detachment of his Majesty's 44th Regiment, on their Voyage to Calcutta, in the Summer of 1822, &c. &c. By RICHARD JONES, Leamington, Member of the Royal College of Surgeons, and late Assistant Surgeon to the Warren Hastings 402

Practical Remarks. Part I. on Acute and Chronic Ophthalmia, Ulcers of the Eye, &c. &c. Part II. on Remittent Fever, viz. Simple and Complicated. By THOMAS O'HALLORAN, M.D. &c. &c. . . . ib.

Observations on the History and Treatment of the Ophthalmia accompanying the Secondary Forms of Lues Venerea. Illustrated by Cases. By THOMAS HEWSON, A.B. Member of the Royal College of Surgeons in Ireland, &c. ib.

Observations on Fever. By R. WADE, Member of the Royal College of Surgeons, and Apothecary to the Westminster General Dispensary . 419

Commentaries on the Diseases of the Stomach and Bowels of Children. By ROBLEY DUNGLISON, M.D. &c. &c. . . . . . 493

Transactions of the Association of Fellows and Licentiates of the King's and Queen's College of Physicians in Ireland. Vol. IV. . . . 504

### FOREIGN.

Consultations et Observations de Medecine, de feu C. L. DUMAS; publiée par le Dr. ROUZET, &c. &c. . . . . . . . 148

Du Froid, et de son Application dans les Maladies; Considerations Physiologiques et Therapeutiques; Observations et Corollaires. Par S. TANCHOU, Docteur en Medecine, &c. . . . . . . . . 249

PAGE

Compètitio ad Aggregationem jussu Regis optimi et ex Mandato summi Regiæ universitatis Magistri, instituta anno 1823. An in Curandi Oculi Suffusione (vulgo Cataracte), Lentis Crystallinæ Extractio hujus Depressione præstantior? Theses quas, Deo favente, in saluberrimâ Facultate medicâ Parisiensi, præsentibus competitiones judicibus, publicis competitorum præsentibus subjiciet et delucidare conabitur die 26 Februarii, anni 1824. Auctor J. Cloquet . . . . . . . . 256

De Medulla Spinali nervisque ex ed producentibus Annotationes Anatomico-physiologicæ. Auctore Carolo Francisco Bellengeri, Regiæ Scientiarum Academiæ et Collegii Medici Taurinensis Membro, &c. &c. . 344

Recherches nouvelles et Observations pratiques sur le Croup, et sur la Coqueluche, suivies de Considerations sur plusieurs Maladies de la Poitrine et du Conduit de la Respiration, dans l'Enfance et dans la Jeunesse. Par Theodore Guibert, Docteur en Medecine, Paris . . . 426

Memoire sur la Neurite Puerperale, ou Inflammation des Nerfs chez les Femmes en Couches, d'aprés des Observations de la Maternité. Par M. A. Duges . . . . . . . . . 516

### STATISTICAL MEDICINE.

Medical Report, laid before the Annual General Meeting of the Governors of the Hospital for Small-pox, held June 3d, 1824. By G. Gregory, M.D. 170

Return of the Number of Recruits for the Army, inspected at Glasgow; from the 1st January, 1817, to the 20th June, 1823, divided into annual periods; wherein the Number deemed fit for the Service are distinguished from those considered to be unfit, with the Causes of Rejection . . 172

Influence of Vaccination upon the Mortality of Berlin . . . 532

### MISCELLANEOUS.

Monument to Dr. Baillie . . . . . . . . . 93

New Society of Physicians . . . . . . . 179, 267

Question of Infanticide , . . . . . . . . 264

New Regulations in the University of Edinburgh . . : . 353

Medical, Clerical, and General Life Assurance Company . . . 357

New Regulations at Apothecaries' Hall . . . . . 532

### OBITUARY.

Mr. Chevalier . . . . . . . . . 93

### MONTHLY CATALOGUE OF BOOKS.

Pages 181, 269, 357, 445, 534.

### METEOROLOGICAL TABLES.

Pages 94, 182, 270, 358, 446, 533.

### NOTICES TO CORRESPONDENTS.

Pages 94, 182, 270, 446, 534.

### ERRATA.

Pages 270, 358, 534.

### NAMES OF AUTHORS,

*Of whose Works, Observations, &c. either a detailed Account, or more or less general View, is given in*

### THE FIFTY-SECOND VOLUME OF THE LONDON MEDICAL AND PHYSICAL JOURNAL.

PAGE

Abercrombie, Dr. on Diseases of the Heart . . . 33

———— on Diseases of the Stomach . . . 41

Amusat, M. on the Structure of the Gall-bladder . . . 258

Andral, Dr. jun. on Rupture of the Heart . . . 175

———— on Alterations of the Bronchiæ . . . ib.

Bailly, M. on the Nervous System . . . 11

Balfour, Dr. on Percussion and Compression . . 104, 200, 284

———— on Hernia . . . 378

Balingall, Dr. on Syphilis . . . 79

Bardsley, Dr. on Pumping in cases of Lunacy . . . 443

Barnes, Dr. account of a Man who swallowed a Table-knife . . . 397

Baudelocque, M. Substitute for the Cæsarean Operation . . . 436

Bayle, M. cases of Diseased Brain . . . 30

———— case of Rupture of the Heart . . . 528

Beatty, Dr. on Pyrola Umbellata . . . 62

Beclard, M. case of Extirpation of the Parotid Gland . . . 69

Becquerel, M. on Alteration of Solutions by the contact of Air . . . 179

Begeschi, Dr. Statements relative to the Lying-in Hospital at Florence . 438

Belhomme, M. on the condition of the Blood in Disease . . . 43

Bellengeri, Dr. on the Functions of certain Nerves . . . 15

Blackmore, Dr. on Enteritis . . . 116

Bory de St. Vincent, M. on a new species of Acaris . . . 527

Boyle, Mr. on Syphilis . . . 78

Brown, Mr. case of spontaneous Evolution of the Fœtus . . . 211

Burdail, Dr. on Mugwort in Epilepsy . . . 531

Bussy, Mr. on Liquifaction of the Sulphureous Acid . . . 178

Buttner, M. on Menstruation from the Mammæ . . . 434

Callow, Mr. case of Formation of Pus in the Stomach . . . 123
———— case of Wound from Dissection . . . 125
Carson, Dr. on the Circulation in the Brain . . . 18
Chevreul, M. on the Blood in Jaundice . . . 258
Combe, Dr. case of Anæmia . . . 51
Crampton, Dr. case of Chorea . . . 129
———— case of Laryngitis . . . 218
Creighton, Mr. on Tartar-emetic Ointment in Epilepsy . . . 306
Cullerier and Maingault, MM. on Ossifications of the Arachnoid Membrane . . . 259

De Capello, Dr. on Hydrophobia . . . 433
Defermon, M. on Contraction of the Spleen . . . 23
Delacour Faneau, M. on Castration . . . 259
Delpech, M. extirpation of a large Tumor . . . 72
Desmoulins, M. on the Brain of Fishes . . . 17
Domanget, M. case of Rupture of the Vena Cava . . . 527
Duncan, Dr. case of Bifid Brain . . . 24
———— on diffuse Inflammation of the Cellular Membrane . . . 40
Dupuytren, M. on Lithotomy . . . 263

PAGE

EDWARDS, Dr. on the Influence of Physical Agents . . . . 20
ELLICK, Mr. his case of Abdominal Abscess . . . . 278
ELLIOTTSON, Dr. on Pulvis Antimonialis . . . . . 64

FARADAY, M. on Fumigation . . . . . . . 490

FELICI, Dr. DE, case of Anasarca with Pregnancy . . . 439

FENOGLIO, Dr. case of Hydrocephalus cured by Puncture . . 261

———— on Croton Oil . . . . . . . 435

FIX, Dr. case of Rupture of the Bladder . . . . . 259

GALNECHE, M. on Hydrocyanic Acid in Tænia . . . . 530

GASPARD, M. on Putrid Diseases . . . . . . . 52

GARTNER, Dr. on a Glandular Structure in the Uterus . . . 5

GERARDIN, M. on the bad effects of the Ergot . . . . 530

GOOGH, Dr. on Uterine Hemorrhage . . . . . . 440

GREEN, Mr. on Fumigation . . . . . . . 387

HANCOCK, Mr. on the Preparation of Caoutchouc . . . . 444

HAHNEMANN, Dr. on Homœopathia . . . . . . 56

———— on Belladonna as a Preventive of Scarlet Fever 57, 262

HARDY, Mr. on Small-pox after Vaccination . . . . 208

HARE, Dr. on artificial Chalybeate Water . . . . . . 178

HARRISON, Mr. on the Anatomy of Arteries . . . . 9

HARROLD, Mr. on Mr. Earle's Fracture-bed . . . . 375

HELLER, Dr. on Hydrocyanic Acid . . . . . 63, 262

———— spontaneous Cure of Palsy . . . . . 352

HOLMES, Dr. on Malformation . . . . . . . 34

HORNER, Dr. on a new Muscle of the Eye . . . . 2

HUFELAND, Dr. on a Substitute for Castor Oil . . . . 531

HUTCHINSON, Mr. on Neuralgia . . . . . . . 281

JACOBSON, Dr. on the Supra-renal Capsules . . . . . 7
JONES, Dr. on Inflammation of the Spinal Marrow . . . 17

KENNEDY, Dr. case of Gangrene of the Heart . . . . 35

KEY, Mr. on Lithotomy . . . . . . . . 80

KINGLAKE, Dr. on After-pains . . . . . . . 291

———— on Hernia . . . . . . . . 369

———— on Snuff-taking . . . . . . . 481

KOREFF, M. on Pustules under the Tongue in Hydrophobia . . 526

LALLEMAND, M. case of Amputation of the Lower Jaw . . . 68

LARREY, Baron, on the Nerves . . . . . . . 15

LASSAIGNE, M. on the detection of Hydrocyanic Acid in the Body . 263

LEVEILLE, M. on Wasting of the Optic Nerves . . . . . 524

LIAUBON, M. on Salt correcting the Fetor of Cancer . . . 531

LISFRANC, M. on Amputation at the Shoulder-joint . . . 76

———— on the application of the Stethoscope to Fractures . . 77

LISTON, Mr. on Amputation . . . . . . . . 74

MACARTNEY, Dr. on Curvatures of the Spine . . . . 359

MACLEAN, Sir L. on Dropsy . . . . . . . 447

MAGENDIE, M. on the Nerves . . . . . . . 14

———— his Experiments on the Nerves . . . . 82

MARTINET, M. on the Oil of Turpentine as a remedy in Neuralgia . 62

MAURY, Dr. Experiments on the Itch . . . . . . 437

MAXWELL, Dr. on Obstruction of the Bowels . . . . 61

MECKEL, M. his System of Comparative Anatomy . . . . 8

MELIN, Mr. Report of Ocular Diseases . . . . . 183, 271

MEYERS, Dr. on Cynanche Trachealis . . . . . 298

MILLET, Dr. case of a Wound through the Diaphragm . . . 260

MONEY, Mr. case of chronic Hydrocephalus . . . . 462

PAGE

MOORE, Mr. on Pulvis Antimonialis . . . . . . 177

MOREAU DE JONNES, on the Cholera of India . . . . 176

MORIN, M. Analysis of the Male Fern-root . . . . 264

NAUCHE, Mr. on Diagnosis in Disease of the Bronchiæ . . . 432
NORTH, Mr. on certain Appearances in the Stomach . . . 457

OLLIVIER, Dr. cases of Interruption of the Spinal Chord . . . 31
OZANAM, Dr. case of Tuberculous Affection of the Brain . . . 529

PELLETIER and CAVENTOU, MM. on the Analysis of the Upas . 264, 442
POALI, Dr. on Exhalation of Water frum the Lungs . . . 176
PRIOU, M. on Nitiate of Silver in Chorea . . . . . 262

QUADRI, Dr. case of Diplopia . . . . . . 178

REID, Dr. on Epilepsy . . . . . . . . 309
RIBES, Dr. on the Anatomy of the Ear . . . . . . 3
RICHERAND and CLOQUET, MM. on the Elongation of the Extremities . 260
RICHOND, M. on the Influence of the Stomach in producing Apoplexy . 350
RITCHIE, Mr. on the Sex of the Chick in Ovo . . . . 486
ROYER, M. on Digitaline . . . . . . . 441

SAMSON, M. on Death from the Introduction of Air into the Veins . . 525
SANDERS, Mr. case of Puerperal Fever . . . . . 214
——————— case of Ascites during Pregnancy . . . . 302
SARDHAM, Mr. on Cholera . . . . . . . . 295
SCOUTTETEN, Dr. on the Pathological Anatomy of the Peritoneum . 37
SCUDAMORE, Dr. on the Blood . . . . . . . 45
SEWEL, Mr. on contagious Glanders . . . . . . 444
SMITH, Dr. GORDON, on Infanticide . . . . . 264
ST. HILAIRE, M. GEOFFROY, on the Generative System of the Ornythohyncus 6
STOKER, Dr. on the Blood . . . . . . . . 45
SYME, Mr. on Amputation at the Hip-joint . . . . . 66
SHAW, Mr. Remarks on Magendie's Experiments . . . . 95

TANTINI, Professor, Experiments on Vomiting . . . . 524
TONELLI, Dr. on Tartar-emetic Ointment . . . . . 434
TREVIRANUS, Dr. on the Brain . . . . . . . 9
TULLY, Mr. on the Topography of Malta . . . . . 469
THOMAS, Mr. on Sudden Parturition . . . . . 353
TELESIUS, Dr. on the Effects of Lightning . . . . . 393

VANDERBURGH, Dr. case of Chorea . . . . . . 216
——————— cases of Neuralgia . . . . . 373
VENABLES, Mr. on Hydrocephalus . . . . . . 191
VELPEAU, M. on Phlegmasia Dolens . . . . . . 434

WHITE, Mr. case of Lithotomy performed on a Horse . . . 304
WOOLASTON, Dr. on Semi-decussation of the Optic Nerves . . 390

YEATMAN, Mr. case of Malformation . . . . . . 367

ZACCHARI, Dr. on Nitrate of Potash in Hemorrhage . . . 531

### NOTICE TO CORRESPONDENTS.

*In our anxiety to render the* Historical Retrospect *as complete as possible, we have encroached so much upon our limits, as to be obliged to add two additional Half-sheets, and to print the whole in a smaller type than usual. We have likewise judged it expedient to give an account of* M. Magendie's *Experiments, while the interest occasioned by their performance in London remains fresh: we have thus been obliged to omit all the other Departments, but the usual Arrangement will be resumed next Month.*

*Numerous Papers have been received, which shall appear in the ensuing Number.—The* List of Books *is unavoidably postponed.*

